# A scoping review of proton radiation therapy and mutant-isocitrate dehydrogenase-inhibitors in IDH mutated lower-grade gliomas: pushing beyond surrogate end-points

**DOI:** 10.1007/s00701-025-06612-6

**Published:** 2025-07-19

**Authors:** Dima Harba, Alba Corell, Alireza Mansouri, Petter Brandal, Malin Blomstrand, Asgeir Store Jakola

**Affiliations:** 1 Institute of Neuroscience and Physiology, Section of Clinical Neuroscience, Sahlgrenska Academy, Gothenburg, Sweden; 2 Department of Neurosurgery, Sahlgrenska University Hospital, Gothenburg, 413 45 Sweden; 3 Department of Neurosurgery, Penn State College of Medicine, Hershey, PA United States; 4 Department of Oncology, Oslo University Hospital, Oslo, Norway; 5 Institute for Clinical Medicine, University of Oslo, Oslo, Norway; 6 Department of Oncology, Sahlgrenska University Hospital, Gothenburg, Sweden; 7 Department of Oncology, Institute of Clinical Sciences, University of Gothenburg, Gothenburg, Sweden

**Keywords:** Proton, IDH inhibitor, Vorasidenib, Survival, Quality of life, Glioma

## Abstract

**Background:**

Proton radiation therapy (PRT) and mutant isocitrate dehydrogenase inhibitors (mIDH-inhibitors) are emerging therapies for mIDH lower grade gliomas (LGGs). Despite their substantial theoretical benefits, comparisons with current standards – particularly pertaining to patient-centred outcomes – are limited.

**Methods:**

Through PubMed and Scopus, a search strategy based on keywords focusing on PRT and mIDH-inhibitors was applied on December 3, 2024. Studies in English on at least 20 adult patients (≥ 18 years) with mIDH-LGG grade 2 or 3 and published between January 1, 2011 and August 31, 2024 were included. Review articles were excluded.

**Results:**

Of 6383 identified articles, seven per treatment strategy were included. Overall survival was not reported for mIDH-inhibitors. The lack of high-quality studies comparing PRT to photon radiation therapy precludes conclusions regarding efficacy, effectiveness or even post-PRT radiological manifestations. For the mIDH-inhibitor Vorasidenib (AG-881), the radiological objective response rate was 10.0–42.9%, although lower for contrast-enhancing tumors. Vorasidenib significantly delayed tumor progression (27.7 versus 11.1 months, *p* < 0.001) and time to next intervention (not reached versus 17.8 months, *p* < 0.001) when compared to placebo. Adverse events were mostly mild, including elevated liver enzymes (15.6–44.2%) and headache (26.9–46.2%). Only 1/14 studies included assessments related to quality of life (QoL)-domains with inconclusive research on cognitive outcomes.

**Conclusion:**

Studies reporting on patient-centred data including survival, cognition and QoL remain scarce. Larger, comparative prospective studies, preferably randomized controlled trials, with such outcomes are needed to inform clinicians whether the theoretical and radiological benefits can be translated to improved outcomes that matter to patients, i.e. living better and/or longer.

**Supplementary information:**

The online version contains supplementary material available at 10.1007/s00701-025-06612-6.

## Introduction

Isocitrate dehydrogenase (IDH)-mutant lower grade gliomas (LGGs) are slow growing, infiltrating brain tumors. With new treatments emerging, longer survival of affected patients is anticipated [4, 23, 25, 53]. Therefore, finding effective tumor therapies while simultaneously preserving a good quality of life (QoL) is a priority [51].

Surgical resection is often incomplete and both acute and late side effects are a concern after chemo- and/or radiotherapy [7]. Patients not only face shortened survival but also risk tumor- and treatment-related adverse effects [13, 26, 35, 61]. Nevertheless, current optimism in the field of LGG is present due to emerging therapies, namely proton radiation therapy (PRT) and mutant IDH-inhibitors (mIDH-inhibitors).

PRT holds promise to reduce radiation-related side effects given the beneficial dose-delivery to the healthy brain. Contrary to photons, protons have a determinate range and deposit most of their energy just as they come to a stop in the tissues at the so-called Bragg peak, thereafter, showing a steep dose fall-off. This abrupt stop helps to avoid the widespread low-dose radiation to healthy brain tissue [60].

mIDH-inhibitors such as Vorasidenib can halt the disease owing to their ability to decrease the elevated levels of the oncometabolite D-2-Hydroxyglutarate (2-HG) thereby facilitating cell differentiation, immune cell activation and decreasing tumor proliferation [9, 30, 31, 39].

These emerging therapies, both with a solid theoretical fundament, are increasingly used and reported in patients with mIDH-LGG. Before these approaches are universally or even widely used, it is important to illuminate their impact on aspects that matter to patients. Patients are typically concerned if their survival, cognition or QoL will be impaired. Similarly, patients without access to these treatments may wonder what they are missing out on. In this scoping review, we present the available clinical literature and emphasize the lack of studies demonstrating effects in these patient-centred aspects. Further, we discuss the importance of pushing for evidence beyond surrogate end-points also in slow-growing neoplasms like mIDH-LGGs.

The aim of this scoping review was to investigate the current clinical literature on the demonstrated effects of PRT and mIDH-inhibitors with regards to *patient-centred outcomes,* including improved survival, QoL or spared cognitive function, in adult patients diagnosed with mIDH-LGG grade 2 or 3.

## Methods

### Search strategy

Due to the expected limited, heterogenous literature along with the fair recency of these treatments in a group of patients requiring long follow-ups, a scoping review was deemed the most appropriate methodology in order to evaluate the current standing and potentially illuminate gaps in the literature where the need of future research can be directed. This scoping review was conducted, and results reported in alignment with guidelines from Preferred Reporting Items for Systematic reviews and Meta-Analyses extension for Scoping Reviews (PRISMA-ScR) [62]. Relevant articles were identified by searching the databases PubMed and Scopus. Assisted by a trained librarian, two search-blocks were formulated based on terms and keywords used in the Medical Subject Headings (MeSH) thesaurus and existing literature on PRT and mIDH-inhibitors (see Supplementary Information 1 for full search-blocks (Online Resource 1)). The search was performed on December 3, 2024.

### Eligibility criteria

Retrospective and prospective studies where patients with mIDH-LGG grade 2 or 3 received PRT and/or mIDH-inhibitors were included. Only papers written in English with a sample size of at least 20 adult patients (≥ 18 years old) were considered. Studies with at least 20 total patients of which ≥ 80% of patients had mIDH-tumors of grades 2 or 3 were accepted in studies with mixed populations. The inclusion period was between January 1, 2011, and August 31, 2024, in order to allow some years to pass since the identification of IDH in gliomas [42]. Review articles, studies focusing on laboratory research including animal or studies primarily in pediatric populations were excluded.

### Study selection and presentation

**Fig. 1:**
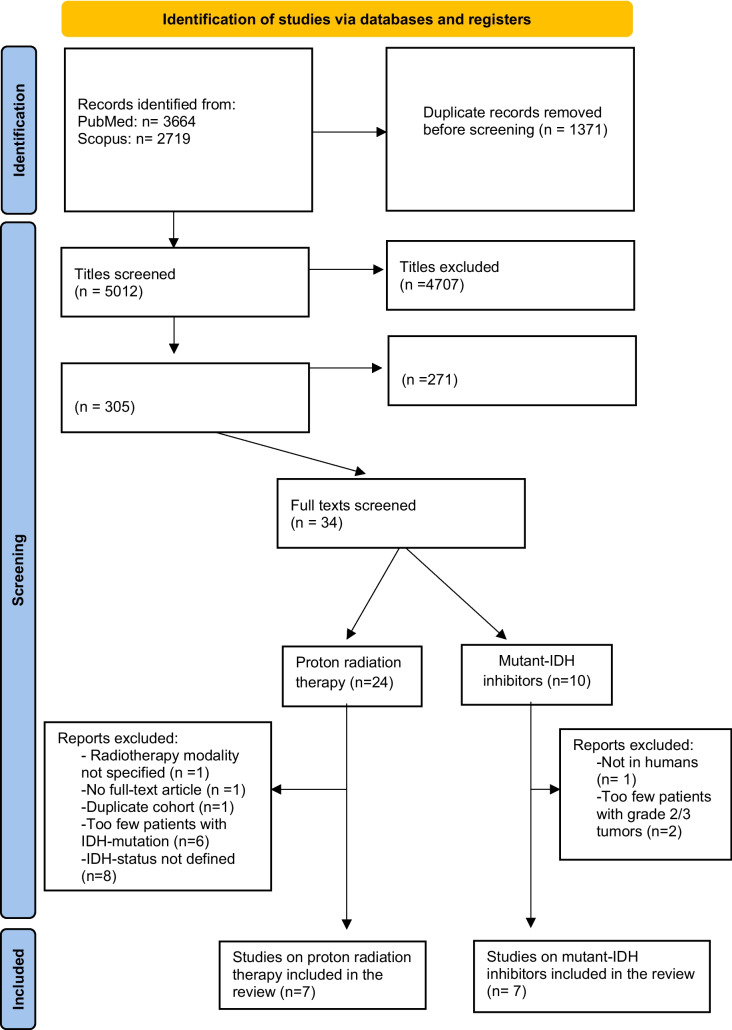
PRISMA flow diagram showing inclusion of studies, IDH = Isocitrate dehydrogenase

Titles and abstracts were initially screened by two independent reviewers (AC and DH). Full-text assessments were made thereafter to ensure eligibility. A third reviewer (ASJ) was consulted when eligibility was doubted. As limited eligible literature was anticipated, no meta-analysis was performed. A PRISMA flowchart (see Fig. 1) was used to demonstrate identification and inclusion of the studies [41]. Through tabulation, the data extracted from included full-text articles was presented in Table 1 for PRT and Table 2 for mIDH-inhibitors.

**Table 1:** Characteristics of studies concerning PRT, n:7

				Number of patients				Main Findings in relation to PRT			
Study	Design	Follow-up: Median (range) months	Median Dose, Gy RBE (range)	Total, n	Tumor Grade (G)	IDH-status, n (%)	Aim	Survival	QoL	Toxicities	Radiological manifestations
Acharya et al. (2018) [1]	R	PRT: 19.6 (9.7–31.0) Photon: 34.5 (2.8–109.7)	PRT: 54.0 (50.4–59.4) Photon: 59.4 (50.4–63.0)	Total:160 PRT: 37 Photon:123	PRT: G2/3: n:19/18 %: 51/49 Photon: G2/3: n: 48/75 %: 39/61	PRT: -mIDH: 31(84) Photon: -mIDH: 73 (59) -Unknown: 22 (17)	Identify risk factors and incidence of developing clinically significant RN in PRT vs. photon treated gliomas	NR	NR	NR	2-year cumulative incidence for RN: -PRT vs. photon: 18.6% vs. 9.9% - Oligodendroglioma/Astrocytoma: 24.2%/6.2%
Dworkin et al. (2018) [14]	R	Overall: 58.0 (6.0–130.0) PRT + TMZ: 35.0 (NR) PRT only: 64.0 (NR)	PRT only: 54.0 (45.0–59.4) PRT + concurrent/adjuv-ant TMZ: 56.1 (54.0–60.0) PRT + adjuvant TMZ: 59.4 (54.0–59.4)	Total: 119 PRT + TMZ: 33 PRT only: 86	G2/3: n: 81/38 %: 62/29	mIDH: 81 (68)	Investigate whether administering PRT + TMZ vs. only PRT increases risk of PsP development	-Worse OS and FFP with larger target volumes -Better OS and FFP after 24 months with PsP presence	NR	NR	Increased risk for PsP with: -PRT + TMZ (hazard ratio:2.2) -G3 mIDH-tumors (63% vs. 35% in G2 tumors)
Eichkorn et al. (2023) [15]	R	PRT: 5.1 years (7 months-11 years)	Overall: 54.0 (50.4–60.0) G2: 54.0 (50.4–60.0) G3: 60.0 (54.0–60.0)	Total: 194	G2/3: n:128/66 %:66/34	mIDH: 194 (100)	Evaluate the efficacy, safety and long-term clinical and imaging outcomes of PRT in mIDH G2 and G3 gliomas	5-year OS: G2/3: %: 85/67 5-year PFS: G2/3: %: 60/30	NR	-Acute toxicities, CTCAE < 3^a^: Fatigue, Headache, Erythema, Focal alopecia -No CTCAE ≥ 3 nor late toxicities	Overall RICE: 25% of the cohort, of which 31% symptomatic and 35% required therapy RICE related to grade, G2/G3: %: 29/17 RICE: No effect on OS or PFS
Ek et al. (2023) [17]	R	*Mean follow-up.:* PRT: 26.0 (20.0–31.0) Photon: 69.0 (62.0–77.0)	PRT: 54.0 (54.0–60.0) Photon:59.0 (54.0–64.0)	PRT: 44 Photon: 98	PRT G2/3: n: 29/15 %: 66/34 Photon G2/3: n: 40/58 %: 41/59	PRT: -mIDH: 36 (82) Photon: -mIDH: 26 (27) -Unknown: 57 (58)	Compare treatment outcomes, patient characteristics and survival between PRT and photon therapy	Decreased PFS and OS with higher average doses Median PFS and OS not reached	NR	Acute alopecia CTCAE grade 2: PRT: 15.9% vs. Photon: 5.1% No difference in memory impairment in PRT vs. photon Less acute fatigue CTCAE grade 2 with PRT	PsP: -Total: n: 21/126 -PRT: n:4, 3% -Photon: n:17, 13%
Gómez Vecchio et al. (2024) [19]	P	PRT: 12.0 (NR) Photon: 12.0 (NR)	NR	Total: 51 PRT: 32 Photon: 6	G2/3: n: 33/18 %: 65/35	mIDH: 51 (100)	Report on and find predictors of global health status and fatigue changes from preoperative setting to 12 month postoperatively	NR	PRT: Less unfavourable changes in fatigue No difference in global health status (PRT vs photon)	NR	NR
Qiu et al. (2022) [47]	R	PRT: 21.7 (NR)	G2: 54.0 (54.0) G3: 60.0 (60.0) G4: 60.0 (54.0–60.0)	PRT: 52	G2/3/4: n: 22/25/5 %: 42/48/10	mIDH: 52 (100)	Outcomes of PRT in mIDH gliomas	PFS: 12/24 months: 97.6%/78.4% OS: 12/24 months: 100%/91% Tumor grade significantly impacted both PFS and OS	NR	All acute toxicities: n:40 CTCAE grade 1, Alopecia, n:38 Late toxicities: n:9, CTCAE grade 2 and 3, n:5 including: Dizziness, Fatigue, Memory impairment, Epilepsy	RN:1.9%; observed in oligodendroglio-ma G3 only
Ritterbusch et al. (2021) [50]	R	PRT: 32.0 (17.0–57.0) Photon: NR	53.9 (42.0–60.0)	PRT: 57 Photon: 43	PRT G2/3: n:21/36 %: 37/63 Photon G2/3: n:12/31 %: 28/72	PRT: -mIDH: 49 (86) Photon: -mIDH: 24 (56) -Unknown: 8 (19)	To distinguish patterns of PsP post-PRT vs PsP after photon therapy	No difference in OS between: -Patients with/out PsP post-PRT -PRT and photon	NR	Asymptomatic PsP: n:5/14 Symptomatic PsP: n:9/14, including vision loss, memory loss	PsP: -PRT: n:14, 24.6% of which n:9, 64% were symptomatic -Photon: NR

*CTCAE*; Common terminology criteria for adverse events, *FFP*; Freedom from progression, *G*; Tumor Grade, *Gy*; Gray, *IDH*; Isocitrate dehydrogenase, *mIDH*; mutant- isocitrate dehydrogenase, *NR*; Not reported, *OS*; Overall survival, *P*; Prospective, *PFS*; Progression Free Survival, *PRT*; Proton Radiation Therapy, *PsP*; Pseudoprogression, *QoL*; Quality of Life, *R*; Retrospective, *RBE*; Relative biological effectiveness, *RICE*; Radiation-induced Contrast Enhancement, *RN*; Radiation necrosis, *TMZ*; Temozolomide, *vs.*; versus

^a^Rates unspecified for each adverse event

**Table 2:** Characteristics of studies on mIDH-inhibitors, n:7

				Number of patients				Main Findings in relation to mIDH-inhibitor administration			
Study	Design	mIDH-inhibitor	Median treatment duration (range)	Total, n	Tumor Grade (G),	Previous therapy, n (%)	Aim	Survival	QoL	Toxicities	Treatment Response/*ORR (%)*
Cho et al. (2022) [9]	R	-Ivosidenib (AG-120) -Vorasidenib (AG-881)	*Follow-up:* Up to 4 months post-treatment	Ivosidenib: 18 Vorasidenib: 11	G2/3/4: n:19/7/3 %: 66/24/10	NR	To investigate MRI changes in gliomas during mIDH-inhibitor treatment at 3–6 weeks and/or 2–4 months after treatment start	NA	NR	NR	*Radiological observations:* At 3–6 weeks: Increase in nrCBV and median nrCBV/ADC but not in ADC or FLAIR At 2–4 months: No changes from baseline
Mellinghoff et al. (2021) [32]	P, Open-label	Vorasidenib (AG-881)	Enhancing: 3.3 months (0.2–53.6) Non-enhancing: 26.8 months (1.0–50.9)	Total Glioma: 52 Enhancing: 30 Non-enhancing: 22	G2/3/4: n:25/22/4 %: 48/42/8 Unknown: n:1 %: 2	Enhancing: -RT: 22 (73) -Systemic: 25 (83) -Only Surgery: 4 (13) Non-enhancing: -RT: 8 (36) -Systemic: 14 (64) -Only surgery: 7 (32)	Investigate safety and outcomes of Vorasidenib	Median PFS: -Overall Cohort: 7.5 months -Enhancing: 3.6 months -Non-enhancing: 36.8 months	NR	Any CTCAE, n: 52, most common: Headache 46.2% ALT elevation 44.2% CTCAE ≥ 3, n:10 ALT/AST elevation, Fatigue, Nausea, Seizure, Vomiting, Decreased neutrophil count	Enhancing: ORR (0.0) Non-enhancing: ORR (18.2)
Mellinghoff et al. (2023) [30]	RCT, Open-label	Ivosidenib (AG-120) Vorasidenib (AG-881)	Vorasidenib: 14.3 months (0.9 −22.6) Ivosidenib: 15.1 months (1.8–22.1)	Vorasidenib: 24 Ivosidenib: 25	Vorasidenib: G2/3: n: 22/2 %: 92/8 Ivosidenib: G2/3: n:21/4 %: 84/16	Vorasidenib:-Surgery:24 (100)-RT:7 (29)-Systemic:10 (42)Ivosidenib:-Surgery:25 (100)-RT: 7 (28)-Systemic: 14 (56)	To investigate the mechanism of action of Vorasidenib and Ivosidenib	Median PFS not reached in either mIDH-inhibitor	NR	Vorasidenib: -Any CTCAE, n: 24, most common: Nausea, Headache 41.7%, Diarrhea, Fatigue 29.2% -CTCAE ≥ 3, n:7 Anemia, ALT-elevation, hyperglycemia, hypophosphatemia Ivosidenib: -Any CTCAE, n:25, most common: Headache, Anemia 36.0%, Diarrhea, Seizure 28.0% -CTCAE ≥ 3, n:6, hyponatremia	Vorasidenib −50mg q.d.: ORR (42.9) −10mg q.d.: ORR (10.0) Ivosidenib −500 mg q.d.: ORR (35.7) −250 mg b.i.d.: ORR (12.5) 2-HG concentration reduction: -Vorasidenib 50 mg q.d.: 92.6% reduction -Ivosidenib 500 mg q.d.: 91.1% reduction
Mellinghoff et al. (2023) [31]	RCT, Double-blinded	Vorasidenib (AG-881)	*Median follow-up (Interquartile range):* Vorasidenib: 14.0 months (10.1 to 17.9) Placebo: 14.3 months (10.0 to 18.1)	Vorasidenib: 168 Placebo: 163	G2: n:331 %: 100	Surgery: 331 (100)	Investigate PFS and time to next intervention in grade 2 tumors receiving Vorasidenib	Vorasidenib vs placebo: -PFS: 27.7 vs. 11.1 months -Time to next intervention: not reached vs. 17.8 months	NR	-Any CTCAE, n:141, most common: ALT elevation 38.9%, Fatigue 32.3% -CTCAE ≥ 3, n:27, ALT/AST/gamma-glutamyl transferase elevation, Fatigue, Diarrhea, Seizure	Vorasidenib 40 mg q.d.: ORR (10.7) Placebo: ORR (2.5)
Natsume et al. (2022) [39]	P, Open-label	Safusidenib (DS-1001)	Enhancing: 7.3 weeks (0.0–190.0) Non-enhancing 91.2 weeks (15.0–207.0)	Total: 47 Enhancing: 35 Non-enhancing: 12	G2/3/4 n:17/23/7 %36/49/15	RT:47 (100)Chemotherapy:38 (81)-TMZ:35 (75)-Nimusutine14 (30)-Bevacizumab 7 (15)	To determine the safety, efficacy and pharmacology of Safusidenib/ DS-1001	Median PFS Non-enhancing vs. Enhancing: not reached vs.10.4 weeks	NR	-All CTCAE, n:45, most common: Skin hyperpigmentation 53.2% Diarrhea 46.8% -CTCAE 3, n: 20, Diarrhea, Arthralgia, headache, decreased neutrophil count, ALT/AST elevation	-Significantly lower 2-HG in Safusidenib (DS-1001) tumor samples -Enhancing: ORR (17.1) (CR: n: 2, 5.7%, PR: n: 4, 11.4%) -Non-enhancing: ORR (33.3) (PR: n: 1, 8.3%, mR: n: 3, 25.0%)
Peters et al. (2023) [43]	R	Ivosidenib (AG-120)	43.7 weeks (5.5- 74.1)	Total: 30 Enhancing: 8 Non-enhancing: 22	G2/3/4: n:21/8/1 %:70/27/3	RT: 8 (27)TMZ: 18(60)mIDH-peptide vaccine: 2 (7)Bevacizumab1 (3)	To investigate the outcomes of Ivosidenib treatment	Deaths: n:2 (of total 30)	NR	CTCAE 1, n:23, most common: Diarrhea 26.7%, Elevated creatine kinase 33.3%	Enhancing: ORR (0.0) Non-enhancing: ORR (36.3)
Wick et al. (2021) [65]	P	BAY1436032	NR	Total glioma:39 Enhancing: 33 Non-enhancing: 2 Unknown: 4 Dose expansion glioma: 25 Dose escalation glioma: 14	NR	Dose expansion: -RT: 23 (92)Dose escalation:NR separately	To investigate the safety profile and pharmacology of BAY1436032 in mIDH solid tumors	PFS rate at 3 months: 0.31	NR	NA due to analysis with other tumor types	ORR: (11) (CR: n:1, 3.0%, PR: n:3, 9.0%)

*2-HG*; 2-hydroxyglutarate, *ADC*; Apparent diffusion coefficient, *ALT*; alanine aminotransferase, *AST*; aspartate aminotransferase, *b.i.d.*; bis in die, twice a day, *CR*; Complete Response, *CTCAE*; Common terminology criteria for adverse events, *FLAIR*; Fluid-attenuated inversion recovery, *G*; Tumor grade, *mIDH*; mutant-isocitrate dehydrogenase, *mR*; minor Response, *MRI*; Magnetic Resonance Imaging, *NA*; Not Applicable, *NR*; Not reported, *nrCBV*; normalized relative cerebral blood volume, *ORR*; Objective response rate, *P*; Prospective, *PFS*; Progression Free Survival, *PR*; Partial Response, *q.d*.; quaque die, once a day, *QoL*; Quality of Life, *R*; Retrospective, *RCT*; Randomized controlled trial, *RT*; Radiotherapy, *TMZ*; Temozolomide, *vs.*; versus

## Results

Our search identified a total of 6383 articles from PubMed and Scopus with gradual assessments leading to the final inclusion of seven articles per treatment (see Fig. 1).

### Proton radiation therapy

Across all included studies, a total of 535 patients received PRT [1, 14, 15, 17, 19, 47, 50]. All but one article had a retrospective study design, and the single prospective study focused on longitudinal changes in patient-reported outcomes where radiation therapy was a covariate [19]. No eligible randomized controlled trial (RCT) was identified. Median follow-up ranged between 12–64 months and median PRT-dose prescribed was 54–60 Gy RBE across studies. For a detailed overview, see Table 1.

### Survival

While median overall survival (OS) was not reached in two studies, [15, 17], one study reported a progression free survival (PFS) and OS at 24-months after PRT to 78.4% and 91.0%, respectively [47]. In the same study, tumor grade impacted OS and PFS the most as shown by univariate (OS: *p* = 0.002, PFS: *p* = 0.004) and multivariate analysis (OS: *p* = 0.024, PFS: *p* = 0.022) [47]. Another study also indicated the importance of tumor grade, with the 5-year OS in grade 2/3 tumors being 85.0%/67.0%, respectively (*p* = 0.0088) [15]. Similarly, the 5-year PFS in grade 2 and 3 tumors were 60.0% and 30.0%, respectively (*p* < 0.0001) [15].

Two other studies compared survival in patients receiving PRT versus photon radiation therapy [17, 50]. None of the studies showed differences in OS between radiation modalities. Moreover, the proportion of mIDH-tumors in the photon therapy groups in both studies did not satisfy this review’s eligibility criteria.

### Toxicities

Four studies included reports on adverse events [15, 17, 47, 50]. Toxicities mostly occurred early in the post-PRT period and were mild, grading 1–2 on the common terminology criteria for adverse events (CTCAE) (see Table 1). The reporting on specific mild toxicities varied between 1.9% and 73.1%. The most common toxicity was alopecia (15.9–73.1%), but adverse events also included headache, erythema and fatigue. Late, severe toxicities were reported by two studies [47, 50].

The prospective study by Gómez Vecchio et al. indicated a lower proportion of PRT patients experiencing unfavorable changes in fatigue in comparison to photon therapy (66.7% vs. 95.7%, *p* = 0.03) [19]. No differences related to changes in global health status as a QoL-construct were noted (*p* = 0.61) [19]. Another study reported a higher rate of fatigue in photon therapy than PRT up to 3 months posttreatment (*p* = 0.016) thereafter stabilizing in both modalities. The same study reported no difference in subjective memory impairment between radiation modalities [17]. However, the photon therapy group here was not molecularly defined to the same extent, precluding valid conclusions. Other studies reported individual cases developing impaired memory [47, 50].

### Radiological manifestations

Pseudoprogression (PsP) was discussed in three studies [14, 17, 50]. In one study, no difference was found in PsP-rate between patients treated with photons versus protons (*p* = 0.38) [17]. Another study showed PsP in 43.6% (52/119) of PRT-patients with an increased risk of PsP-development in grade 3 versus grade 2 tumors (*p* = 0.003) [14]. However, more grade 2 tumors here had unknown IDH-status. Therefore, caution should be exerted when interpreting these findings [14]. Ritterbusch et al. observed unique imaging patterns of PsP following PRT. There, PsP developed in 24.6% of patients and PsP-lesions were characterized by being small (< 1 cm), transient, multifocal and patchy with a location at the Bragg peak. The mean time to PsP-occurrence following PRT was 15.4 months while photon-related PsP occurred within 3 months [50]. None of the studies showed association between PsP and tumor subtypes [14, 17, 50]. Finally, Eichkorn et al. assessed radiation induced contrast enhancement (RICE), developing in 25.3% of their overall cohort, and in 29.7% of grade 2 versus 16.7% of grade 3 patients (*p* = 0.11) [15]. The presence of RICE did not influence OS (p = 0.15) nor PFS (*p* = 0.67) [15]. However, astrocytomas were associated with earlier RICE development in comparison to oligodendrogliomas (*p* = 0.047) and RICE risk increased independently with older age (*p* = 0.017) [15].

Despite the large proportion of IDH-wildtype tumors in the photon therapy group, Acharya et al. reported the 2-year cumulative incidence for radiation necrosis (RN) following PRT to be 18.6% compared with 9.9% after photon therapy (*p* = 0.16) [1]. Further, the incidence of RN was higher in oligodendrogliomas compared to astrocytomas (*p* = 0.01) with no difference in radiation modality observed (*p* = 0.68) [1]. Another study reported a single case of severe RN (CTCAE grade 3) in their cohort, observed in a patient with oligodendroglioma grade 3 treated with PRT [47].

### Mutant IDH-inhibitors

Out of seven studies on mIDH-inhibitors, two had a retrospective design, [9, 43], while the rest were prospective studies. A total of 73 patients received Ivosidenib (AG-120), 255 received Vorasidenib (AG-881), 47 received Safusidenib (DS-1001), and 39 received BAY1436032. For a detailed overview, see Table 2.

### Progression free survival

None of the seven articles reported on OS while six studies reported PFS [9, 30–32, 39, 65]. In an RCT examining Ivosidenib in non-enhancing LGG, median PFS was not reached after a median postoperative treatment duration of 15.1 months [30]. Another study reported two deaths (of 30 patients in total) after a median time on Ivosidenib of 43.7 weeks [43].

With Vorasidenib, a median PFS of 7.5 months was seen in a broad mIDH glioma cohort, with longer PFS in non-enhancing versus enhancing tumors (36.8 vs. 3.6 months, respectively) [32]. This difference in PFS between enhancing and non-enhancing lesions was seen also with Safusidenib (DS-1001). There, median PFS was 10.4 weeks in enhancing tumors while median PFS was not reached in non-enhancing gliomas after a median treatment duration of 7.3 and 91.2 weeks, respectively [39]. At 3 months, BAY1436032 showed a PFS of 31.0% [65]. The study by Cho et al. included joint data on Ivosidenib and Vorasidenib [9]. A shorter PFS was associated with: 1) continuously decreasing diffusion coefficient on imaging, 2) Fluid-attenuated inversion recovery (FLAIR) volume increase > 4cm^3^ and 3) elevated perfusion values at 2–4 months posttreatment. However, the authors stress not to interpret this as prognostic effects of mIDH-inhibitors, rather as preliminary radiographic observations [9].

Lastly, in the INDIGO double-blinded RCT, Vorasidenib showed significantly prolonged median PFS versus placebo (27.7 vs. 11.1 months; *p* < 0.001) in grade 2 non-enhancing tumors after a median follow-up of 14 months [31]. Similarly, Vorasidenib improved median time to next intervention (not reached vs. 17.8 months, *p* < 0.001) [31].

### Toxicities

Mild toxicities (CTCAE grade < 3) dominated in patients treated with mIDH-inhibitors (see Table 2). With Vorasidenib, headache, nausea and liver enzyme elevation were common. Other infrequent toxicities included diarrhea, constipation and electrolyte and/or glucose disturbances [30–32]. Fatigue was reported at rates around 30% and subjective memory impairment at 16.7% [9, 30–32]. Ivosidenib reported analogous toxicity rates to Vorasidenib, but more electrolyte and/or glucose disturbances and anemia were reported with Ivosidenib. Seizure frequency increased in 13.3% of patients and fatigue constituted 12.0% of all toxicities of Ivosidenib [30, 43].

Safusidenib (DS-1001) exclusively caused frequent dermatological complications in up to 53.2% of patients, alopecia/arthralgia in 27.7% and backaches in 21.3%. Diarrhea was more common while rates of headache and nausea were comparable to other mIDH-inhibitors. Limited electrolyte disturbances or elevation of liver enzymes were noted [39]. Toxicities of BAY1436032 were analysed alongside other tumor types and included diarrhea, nausea, fatigue and liver enzyme elevation [65].

### Therapeutic response

Neither Vorasidenib nor Ivosidenib showed complete responses (CR). In non-enhancing tumors, the highest objective response rate (ORR) was noted following 50 mg Vorasidenib (ORR 42.9%) [30]. The ORR decreased to 10.0% when administering 10 mg Vorasidenib, indicating a dose–response effect. Another Vorasidenib trial showed an ORR of 18.2% in non-enhancing tumors, with a partial response of 4.5% occurring at 50 mg once daily and minor response of 13.6% at 200 mg once daily [32]. No radiographic response was noted in enhancing tumors [32]. The INDIGO trial reported an ORR of 10.7% (vs. 2.5% in placebo) with the administration of 40 mg Vorasidenib once daily [31]. Thus, the range of ORR with Vorasidenib across studies was 10.0–42.9%, at least partially explained by administered doses. Lastly, a 92.6% reduction in 2-HG concentrations in resected tumor tissues was noted in addition to decreased tumor proliferation, more activated immune cells and suppressed genes related to cell-cycle progression. Of note, these analyses were performed in patients who received Vorasidenib 50 mg once daily for 28 (+ 7) days, up until surgery [30].

Ivosidenib also showed better response in non-enhancing versus enhancing tumors (ORR 36.3% vs. 0.0%) [43]. With the administration of 500 mg Ivosidenib once daily, an ORR of 35.7% was reported. The ORR decreased almost threefold with the administration of 250 mg Ivosidenib twice a day instead (ORR 12.5%) [30]. The concentrations of 2-HG in resected tumor tissues were consequently decreased by 91.1% following the administration of 500 mg Ivosidenib once daily for 28 (+ 7) days, up until surgery [30].

Among all mIDH-inhibitors, Safusidenib (DS-1001) reported the highest CR of 5.7% and exclusively in enhancing tumors. Following Safusidenib (DS-1001) administration twice per day in 21-day cycles, the ORR observed was 33.3% in non-enhancing tumors versus 17.1% in enhancing tumors. Further, tumor samples of patients still receiving Safusidenib (DS-1001) but underwent surgery upon progression showed lower 2-HG concentrations after treatment [39]. BAY1436032 specifically targeted enhancing tumors and demonstrated CR in 3.0% of cases [65].

## Discussion

In this scoping review, we demonstrate promising results on surrogate outcomes following both PRT and mIDH-inhibitors. Although the studies involving mIDH-inhibitors were more often prospective and randomized than in PRT, some of the same shortcomings exist. Thus far, no solid evidence exists with regards to patient-centred outcomes such as improved survival, QoL or spared cognitive function following these therapies especially in relation to other treatment options.

### Patient-centred outcomes

To date, only PFS and time to next intervention has been reported for mIDH-inhibitors. Substantial data on OS, QoL and cognition is lacking. Survival comparisons between PRT and photon therapy were limited by the low proportion of mIDH-tumors in the photon groups, often included at an earlier time-point [17, 50]. It is yet to be explored what influences neurocognition in mIDH-LGG; whether it is the disease itself, the treatments applied or a combination of both [11, 58, 59, 67]. Dosimetric studies assessing PRT showed a comparable target volume to photon therapy and delivery of lower radiation doses to organs at risk [2, 22, 68]. This spared critical neurological structures, including areas of neurogenesis (subventricular zone and hippocampus), thereby showing potential in decreasing long term sequelae and improving QoL [2, 22]. Only one included study associated PRT with less unfavourable changes in fatigue affecting QoL [19]. Other included studies reported fatigue, memory impairment and other adverse events without elaborating on specific QoL-domains. Studies not included in this review, assessed QoL with PRT and showed development of neuroendocrine deficiencies with patients retaining cognitive function overtime [55, 57]. In pediatric populations, PRT was associated with less cognitive impairment [20, 33, 38, 45].

### Surrogate end-points

Surrogate end-points applied in this context included PFS and dosimetric advantages. In recent years, cancer drugs showing only a PFS benefit were approved by the Food and Drug Administration [44]. Due to limited therapies for mIDH-LGG, the application of surrogate end-points might accelerate the availability of novel treatments in the market. However, the long-term benefits of these treatments remain unknown and surrogate end-points do not necessarily reflect on outcomes that matter to patients [52, 64].

It remains unclear whether an improved PFS translates into improved survival, QoL or cognition. Deterioration in clinical symptoms does not always correlate with pre-defined radiological progression criteria [34, 52]. A comparable case involved Bevacizumab in glioblastoma-treatment which, despite a beneficial PFS, did *not* ultimately prolong OS nor improve QoL but was more frequently associated with serious toxicities [8, 12, 18]. Similarly, another trial comparing early versus delayed radiotherapy in LGG showed a PFS benefit with early post-surgical radiotherapy that could *not*, however, be translated to improved OS [63]. Even in grade 2 mIDH-glioma, predicting OS or post-progression survival based upon PFS has proven difficult [52]. The dosimetric advantages of PRT may be offset by post-PRT manifestations such as PsP, RICE or RN, especially as these per se may be difficult to differentiate from true tumor progression, thereby leading to unnecessary, and sometimes deleterious, interventions due to misdiagnosis [1, 16, 24, 40, 59]. Perhaps assessing clinical outcomes could better guide treatment decision-making [52].

### Post-PRT radiological manifestations

Contrast-enhancing brain lesions, RN, PsP and RICE were apparent in several included studies [1, 14, 15, 17, 47, 50]. However, the heterogeneity of the results, populations and terminology used to describe these manifestations complicates comparisons and prevents conclusions from being drawn [17, 27, 29]. These inconsistencies in reporting were even apparent in other studies not included in this review.

In similarity to the included study by Acharya et al. [1], another study, not included in this review according to the eligibility criteria, reported a higher proportion of tissue necrosis following PRT compared to photon therapy (18% vs. 6% respectively) [66]. However, consistent with findings from another included study [17], Bronk et al. observed no difference in PsP rate between radiation modalities [6].

Among the included studies, PsP was more frequently observed in grade 3 tumors while RICE rate was higher in grade 2 tumors [14, 15]. PsP was not associated with tumor subtypes [14, 17, 50]. However, astrocytomas showed earlier RICE development, while oligodendroglioma patients -regardless of treatment modality- expressed a higher incidence of RN with one case of severe RN following PRT in grade 3 oligodendroglioma [1, 15, 47]. Despite not meeting this review’s eligibility criteria, Bronk et al. showed earlier occurrence of PsP in oligodendroglioma following PRT and no correlation between PsP rate and tumor grade [6]. In another photon-dominated cohort, all RN cases following PRT occurred in patients with oligodendroglioma [3]. However, too few patients were present in both studies to be able to conclude on this much debated topic [3, 6]. Older age was shown to be an independent risk factor for RICE in one included study [15]. While oligodendrogliomas usually present at higher median ages [25], another study showed slightly higher RICE rate in astrocytomas (50% vs. 46% in oligodendrogliomas) [16]. Hence, inconclusive research is currently present regarding associations between tumor subtype and post-PRT manifestations.

### Limitations

Our search was dominated by single-institutional, retrospective, uncontrolled studies with small sample sizes, mixed IDH-mutational status and short follow-ups. Nonetheless, this reflects the present state of evidence. The latest 2021 World Health Organization classification of tumors was not always applied [28]. For PRT, selection was biased to younger patients, less pre-radiation cognitive impairment and lower tumor grades with lower administered doses in accordance with the current guidelines in order to minimize neurocognitive effects [1, 17, 19]. Further, inconsistent definitions and different nomenclature used to describe post-PRT radiological manifestations prevents current comparisons between proton studies further complicating the clinical application of these manifestations and the gathering of results on common ground [17, 27, 29].

For mIDH-inhibitors, prior therapies and short follow-ups complicate effects on survival, while placebo cross-over in the INDIGO-trial will complicate long term survival analysis. In the INDIGO trial, patients receiving placebo could cross-over to Vorasidenib upon progression. Given the aim to delay radio-chemotherapy, the threshold to receive Vorasidenib as second-line therapy for placebo patients was probably lower than to receive radio-chemotherapy, which was the option in the Vorasidenib arm of this trial [31]. Further, the INDIGO trial compared treatment-naïve patients receiving Vorasidenib monotherapy to placebo [31]. This may limit comparisons to the current standards of LGG treatment. On the other hand, it provides the opportunity for studying a watch-and-wait strategy that is applied in selected centres following surgical resection and compare it with active treatment with presumably less neurotoxic effects [5, 13, 31, 32].

Despite the current caveats, the cumulative evidence suggests that lower-grade, non-enhancing tumors are responsive to mIDH-inhibitors with regards to progression and metabolic alterations such as reduced 2-HG concentrations. For mIDH-LGGs having undergone malignant transformation, studies incorporating combination therapies and trials in enhancing tumors are coveted. Previous studies in acute myeloid leukemia showed significant advantage when mIDH-inhibitors were combined with the hypomethylating agent, Azacitidine [10, 37]. Interestingly, Safusidenib (DS-1001) and BAY1436032 produced CR in *enhancing* tumors, even providing preliminary prospects for systematic targeted therapy in mIDH gliomas of higher grade as well [39, 65].

### Future directions

Assessing OS in mIDH-LGGs might take decades, but relying on surrogate end-points is only a short-term solution. Given the long-expected survival of patients, often undergoing multiple interventions over time, therapies directed at the early stages of disease should prove long-term benefits [52]. Prospective studies with longer follow-ups examining patient-centred domains are, therefore, highly encouraged. Awaiting us in the field of PRT in mIDH-glioma are three prospective, randomized trials; PRO-GLIO, NRG-BN005, and APPROACH; leveraging on survival and cognitive outcomes [21, 23, 56]. Additionally, the production of consensus-based definitions may ease the reporting, analysis and application of post-PRT radiological changes in future studies, a work that has already been started by the European Particle Therapy Network [27]. The long-term OS, cognitive and QoL analyses from the INDIGO trial and further trials on Safusidenib (DS-1001) and Vorasidenib through the VIGOR trial are even forecasted [31, 46, 54]. Finally, to facilitate the holistic reporting and analysis of trial outcomes aligning with patient concerns and priorities, the development of core outcome sets in future trials concerning these treatments is worth considering. So far for adult gliomas, the COBra-study developed a core outcome set where some of the main outcome domains included survival, health-related QoL and cognitive function [36, 48, 49].

## Conclusion

PRT and mIDH-inhibitors are two therapies promising precision in targeting mIDH-LGG. Current reports lack profound assessments of patient-centred outcomes despite their prognostic importance in the young, affected patient group. Prospective, patient-centred studies, preferably RCTs, with longer follow-ups and larger populations, are strongly encouraged to aid treatment decisions.

## Supplementary information

Below is the link to the electronic supplementary material.

ESM 1 (PDF 97.0 KB)

## Data Availability

No datasets were generated or analysed during the current study.

## Abbreviations

2-HG: D-2-Hydroxyglutarate
CR: Complete Response
CTCAE: Common terminology criteria for adverse events
IDH: Isocitrate-dehydrogenase
LGGs: Lower grade gliomas
mIDH-inhibitors: Mutant isocitrate-dehydrogenase-inhibitors
ORR: Objective response rate
OS: Overall survival
PFS: Progression free survival
PRT: Proton radiation therapy
PsP: Pseudoprogression
QoL: Quality of life
RCT: Randomized controlled trial
RICE: Radiation induced contrast enhancement
RN: Radiation necrosis

## References

[1] Acharya S, Robinson CG, Michalski JM, Mullen D, DeWees TA, Campian JL, Chundury A, Bottani B, Hallahan DE, Bradley JD, Huang J (2018) Association of 1p/19q codeletion and radiation necrosis in adult cranial Gliomas after proton or photon therapy. Int J Radiat Oncol Biol Phys 101:334–343. 10.1016/j.ijrobp.2018.01.099 29534896 10.1016/j.ijrobp.2018.01.099

[2] Adeberg S, Harrabi SB, Bougatf N, Verma V, Windisch P, Bernhardt D, Combs SE, Herfarth K, Debus J, Rieken S (2018) Dosimetric Comparison of Proton Radiation Therapy, Volumetric Modulated Arc Therapy, and Three-Dimensional Conformal Radiotherapy Based on Intracranial Tumor Location. Cancers (Basel) 10:401 10.3390/cancers10110401 10.3390/cancers10110401 PMC6266019 30373115

[3] Ahmad H, Martin D, Patel SH, Donahue J, Lopes B, Purow B, Schiff D, Fadul CE (2019) Oligodendroglioma confers higher risk of radiation necrosis. J Neurooncol 145:309–319. 10.1007/s11060-019-03297-7 31549281 10.1007/s11060-019-03297-7

[4] Baek C, Laurenge A, Touat M (2024) Advances in the treatment of IDH-mutant gliomas. Curr Opin Neurol 37:708–716. 10.1097/wco.0000000000001316 39253756 10.1097/WCO.0000000000001316

[5] Bhatia A, Moreno R, Reiner AS, Nandakumar S, Walch HS, Thomas TM, Nicklin PJ, Choi Y, Skakodub A, Malani R, Prabhakaran V, Tiwari P, Diaz M, Panageas KS, Mellinghoff IK, Bale TA, Young RJ (2024) Tumor Volume Growth Rates and Doubling Times during Active Surveillance of IDH-mutant Low-Grade Glioma. Clin Cancer Res 30:106–115. 10.1158/1078-0432.Ccr-23-1180 37910594 10.1158/1078-0432.CCR-23-1180 PMC10841595

[6] Bronk JK, Guha-Thakurta N, Allen PK, Mahajan A, Grosshans DR, McGovern SL (2018) Analysis of pseudoprogression after proton or photon therapy of 99 patients with low grade and anaplastic glioma. Clin Transl Radiat Oncol 9:30–34. 10.1016/j.ctro.2018.01.002 29594248 10.1016/j.ctro.2018.01.002 PMC5862685

[7] Capelle L, Fontaine D, Mandonnet E, Taillandier L, Golmard JL, Bauchet L, Pallud J, Peruzzi P, Baron MH, Kujas M, Guyotat J, Guillevin R, Frenay M, Taillibert S, Colin P, Rigau V, Vandenbos F, Pinelli C, Duffau H (2013) Spontaneous and therapeutic prognostic factors in adult hemispheric World Health Organization grade II Gliomas: a series of 1097 cases: clinical article. J Neurosurg 118:1157–1168. 10.3171/2013.1.Jns121 23495881 10.3171/2013.1.JNS121

[8] Chinot OL, Wick W, Mason W, Henriksson R, Saran F, Nishikawa R, Carpentier AF, Hoang-Xuan K, Kavan P, Cernea D, Brandes AA, Hilton M, Abrey L, Cloughesy T (2014) Bevacizumab plus radiotherapy-temozolomide for newly diagnosed glioblastoma. N Engl J Med 370:709–722. 10.1056/NEJMoa1308345 24552318 10.1056/NEJMoa1308345

[9] Cho NS, Hagiwara A, Eldred BSC, Raymond C, Wang C, Sanvito F, Lai A, Nghiemphu P, Salamon N, Steelman L, Hassan I, Cloughesy TF, Ellingson BM (2022) Early volumetric, perfusion, and diffusion MRI changes after mutant Isocitrate Dehydrogenase (IDH) inhibitor treatment in IDH1-mutant Gliomas. Neurooncol Adv 4:vdac124. 10.1093/noajnl/vdac124 36033919 10.1093/noajnl/vdac124 PMC9400453

[10] DiNardo CD, Schuh AC, Stein EM, Montesinos P, Wei AH, de Botton S, Zeidan AM, Fathi AT, Kantarjian HM, Bennett JM, Frattini MG, Martin-Regueira P, Lersch F, Gong J, Hasan M, Vyas P, Döhner H (2021) Enasidenib plus azacitidine versus azacitidine alone in patients with newly diagnosed, mutant-IDH2 acute myeloid leukaemia (AG221-AML-005): a single-arm, phase 1b and randomised, phase 2 trial. Lancet Oncol 22:1597–1608. 10.1016/s1470-2045(21)00494-0 34672961 10.1016/S1470-2045(21)00494-0

[11] Dirven L, Reijneveld JC, Taphoorn MJB, Coens C, El-Badawy SA, Tzuk-Shina T, Bravo-Marques J, Back M, Stalpers LJA, Stupp R, Baumert BG, Seidel C (2019) Impact of Radiation Target Volume on Health-Related Quality of Life in Patients With Low-Grade Glioma in the 2-Year Period Post Treatment: A Secondary Analysis of the EORTC 22033–26033. Int J Radiat Oncol Biol Phys 104:90–100. 10.1016/j.ijrobp.2019.01.003 30716525 10.1016/j.ijrobp.2019.01.003

[12] Dirven L, van den Bent MJ, Bottomley A, van der Meer N, van der Holt B, Vos MJ, Walenkamp AM, Beerepoot LV, Hanse MC, Reijneveld JC, Otten A, de Vos FY, Smits M, Bromberg JE, Taal W, Taphoorn MJ (2015) The impact of bevacizumab on health-related quality of life in patients treated for recurrent glioblastoma: results of the randomised controlled phase 2 BELOB trial. Eur J Cancer 51:1321–1330. 10.1016/j.ejca.2015.03.025 25899986 10.1016/j.ejca.2015.03.025

[13] Douw L, Klein M, Fagel SS, van den Heuvel J, Taphoorn MJ, Aaronson NK, Postma TJ, Vandertop WP, Mooij JJ, Boerman RH, Beute GN, Sluimer JD, Slotman BJ, Reijneveld JC, Heimans JJ (2009) Cognitive and radiological effects of radiotherapy in patients with low-grade glioma: long-term follow-up. Lancet Neurol 8:810–818. 10.1016/s1474-4422(09)70204-2 19665931 10.1016/S1474-4422(09)70204-2

[14] Dworkin M, Mehan W, Niemierko A, Kamran SC, Lamba N, Dietrich J, Martinez-Lage M, Oh KS, Batchelor TT, Wen PY, Loeffler JS, Shih HA (2019) Increase of pseudoprogression and other treatment related effects in low-grade glioma patients treated with proton radiation and temozolomide. J Neurooncol 142:69–77. 10.1007/s11060-018-03063-1 30488294 10.1007/s11060-018-03063-1

[15] Eichkorn T, Lischalk JW, Hörner-Rieber J, Deng M, Meixner E, Krämer A, Hoegen P, Sandrini E, Regnery S, Held T, Harrabi S, Jungk C, Herfarth K, Debus J, König L (2023) Analysis of safety and efficacy of proton radiotherapy for IDH-mutated Glioma WHO grade 2 and 3. J Neurooncol 162:489–501. 10.1007/s11060-022-04217-y 36598613 10.1007/s11060-022-04217-y PMC10227167

[16] Eichkorn T, Lischalk JW, Sandrini E, Meixner E, Regnery S, Held T, Bauer J, Bahn E, Harrabi S, Hörner-Rieber J, Herfarth K, Debus J, König L (2022) Iatrogenic influence on prognosis of radiation-induced contrast enhancements in patients with glioma WHO 1–3 following photon and proton radiotherapy. Radiother Oncol 175:133–143. 10.1016/j.radonc.2022.08.025 36041565 10.1016/j.radonc.2022.08.025

[17] Ek H, Fagerström Kristensen I, Stenberg L, Kinhult S, Benedek H, Ek S, Engelholm SA, Engelholm S, Munck Af Rosenschöld P (2023) Transitioning from conventional photon therapy to proton therapy for primary brain tumors. Acta Oncol 62:391–399. 10.1080/0284186x.2023.2200150 37203198 10.1080/0284186X.2023.2200150

[18] Gilbert MR, Dignam JJ, Armstrong TS, Wefel JS, Blumenthal DT, Vogelbaum MA, Colman H, Chakravarti A, Pugh S, Won M, Jeraj R, Brown PD, Jaeckle KA, Schiff D, Stieber VW, Brachman DG, Werner-Wasik M, Tremont-Lukats IW, Sulman EP, Aldape KD, Curran WJ Jr, Mehta MP (2014) A randomized trial of bevacizumab for newly diagnosed glioblastoma. N Engl J Med 370:699–708. 10.1056/NEJMoa1308573 24552317 10.1056/NEJMoa1308573 PMC4201043

[19] Gómez Vecchio T, Rydén I, Ozanne A, Blomstrand M, Carstam L, Smits A, Jakola AS (2024) Global health status and fatigue score in isocitrate dehydrogenase-mutant diffuse glioma grades 2 and 3: A longitudinal population-based study from surgery to 12-month follow-up. Neurooncol Pract 11:347–357. 10.1093/nop/npae017 38737607 10.1093/nop/npae017 PMC11085849

[20] Greenberger BA, Pulsifer MB, Ebb DH, MacDonald SM, Jones RM, Butler WE, Huang MS, Marcus KJ, Oberg JA, Tarbell NJ, Yock TI (2014) Clinical outcomes and late endocrine, neurocognitive, and visual profiles of proton radiation for pediatric low-grade gliomas. Int J Radiat Oncol Biol Phys 89:1060–1068. 10.1016/j.ijrobp.2014.04.053 25035209 10.1016/j.ijrobp.2014.04.053

[21] Grosshans D, NRG Oncology, National Cancer Institute (NCI) (2017) Proton Beam or Intensity-Modulated Radiation Therapy in Preserving Brain Function in Patients With IDH Mutant Grade II or III Glioma. ClinicalTrials.gov. https://clinicaltrials.gov/study/NCT03180502. Accessed 27 Feb 2025

[22] Harrabi SB, Bougatf N, Mohr A, Haberer T, Herfarth K, Combs SE, Debus J, Adeberg S (2016) Dosimetric advantages of proton therapy over conventional radiotherapy with photons in young patients and adults with low-grade glioma. Strahlenther Onkol 192:759–769. 10.1007/s00066-016-1005-9 27363701 10.1007/s00066-016-1005-9 PMC5080304

[23] Heggebø LC, Borgen IMH, Rylander H, Kiserud C, Nordenmark TH, Hellebust TP, Evensen ME, Gustavsson M, Ramberg C, Sprauten M, Magelssen H, Blakstad H, Moorthy J, Andersson K, Raunert I, Henry T, Moe C, Granlund C, Goplen D, Brekke J, Johannessen TA, Solheim TS, Marienhagen K, Humberset Ø, Bergström P, Agrup M, Dahl L, Gubanski M, Gojon H, Brahme CJ, Rydén I, Jakola AS, Vik-Mo EO, Lie HC, Asphaug L, Hervani M, Kristensen I, Rueegg CS, Olsen IC, Ledal RJ, Degsell E, Werlenius K, Blomstrand M, Brandal P (2023) Investigating survival, quality of life and cognition in PROton versus photon therapy for IDH-mutated diffuse grade 2 and 3 GLIOmas (PRO-GLIO): a randomised controlled trial in Norway and Sweden. BMJ Open 13:e070071. 10.1136/bmjopen-2022-070071 36940951 10.1136/bmjopen-2022-070071 PMC10030923

[24] Heggebø LC, Borgen IMH, Blakstad H, Saxhaug C, Rønning PA, Niehusmann PF, Werlenius K, Blomstrand M, Brandal P (2025) Case report: Pseudoprogression mimicking neoplastic recurrence three months after completion of proton beam therapy for an IDH-mutant astrocytoma CNS WHO grade 3. Front Oncol 15:1397912. 10.3389/fonc.2025.1397912 39949738 10.3389/fonc.2025.1397912 PMC11821596

[25] Hervey-Jumper SL, Zhang Y, Phillips JJ, Morshed RA, Young JS, McCoy L, Lafontaine M, Luks T, Ammanuel S, Kakaizada S, Egladyous A, Gogos A, Villanueva-Meyer J, Shai A, Warrier G, Rice T, Crane J, Wrensch M, Wiencke JK, Daras M, Oberheim Bush NA, Taylor JW, Butowski N, Clarke J, Chang S, Chang E, Aghi M, Theodosopoulos P, McDermott M, Jakola AS, Kavouridis VK, Nawabi N, Solheim O, Smith T, Berger MS, Molinaro AM (2023) Interactive effects of molecular, therapeutic, and patient factors on outcome of diffuse low-grade Glioma. J Clin Oncol 41:2029–2042. 10.1200/jco.21.02929 36599113 10.1200/JCO.21.02929 PMC10082290

[26] Ius T, Isola M, Budai R, Pauletto G, Tomasino B, Fadiga L, Skrap M (2012) Low-grade glioma surgery in eloquent areas: volumetric analysis of extent of resection and its impact on overall survival. A single-institution experience in 190 patients: clinical article. J Neurosurg 117:1039–1052. 10.3171/2012.8.Jns12393 23039150 10.3171/2012.8.JNS12393

[27] Lauwens L, Ribeiro MF, Zegers CML, Høyer M, Alapetite C, Blomstrand M, Calugaru V, Perri DD, Iannalfi A, Lütgendorf-Caucig C, Paulsen F, Postma AA, Romero AM, Timmermann B, Troost EGC, van der Weide HL, Whitfield GA, Harrabi S, Lambrecht M, Eekers DBP (2025) Systematic review of MRI alterations in the brain following proton and photon radiation therapy: Towards a uniform European Particle Therapy Network (EPTN) definition. Radiother Oncol 208:110936. 10.1016/j.radonc.2025.110936 40360047 10.1016/j.radonc.2025.110936

[28] Louis DN, Perry A, Wesseling P, Brat DJ, Cree IA, Figarella-Branger D, Hawkins C, Ng HK, Pfister SM, Reifenberger G, Soffietti R, von Deimling A, Ellison DW (2021) The 2021 WHO Classification of Tumors of the Central Nervous System: a summary. Neuro Oncol 23:1231–1251. 10.1093/neuonc/noab106 34185076 10.1093/neuonc/noab106 PMC8328013

[29] Lu VM, Welby JP, Laack NN, Mahajan A, Daniels DJ (2020) Pseudoprogression after radiation therapies for low grade glioma in children and adults: A systematic review and meta-analysis. Radiother Oncol 142:36–42. 10.1016/j.radonc.2019.07.013 31431375 10.1016/j.radonc.2019.07.013

[30] Mellinghoff IK, Lu M, Wen PY, Taylor JW, Maher EA, Arrillaga-Romany I, Peters KB, Ellingson BM, Rosenblum MK, Chun S, Le K, Tassinari A, Choe S, Toubouti Y, Schoenfeld S, Pandya SS, Hassan I, Steelman L, Clarke JL, Cloughesy TF (2023) Vorasidenib and ivosidenib in IDH1-mutant low-grade Glioma: a randomized, perioperative phase 1 trial. Nat Med 29:615–622. 10.1038/s41591-022-02141-2 36823302 10.1038/s41591-022-02141-2 PMC10313524

[31] Mellinghoff IK, van den Bent MJ, Blumenthal DT, Touat M, Peters KB, Clarke J, Mendez J, Yust-Katz S, Welsh L, Mason WP, Ducray F, Umemura Y, Nabors B, Holdhoff M, Hottinger AF, Arakawa Y, Sepulveda JM, Wick W, Soffietti R, Perry JR, Giglio P, de la Fuente M, Maher EA, Schoenfeld S, Zhao D, Pandya SS, Steelman L, Hassan I, Wen PY, Cloughesy TF (2023) Vorasidenib in IDH1- or IDH2-mutant low-grade Glioma. N Engl J Med 389:589–601. 10.1056/NEJMoa2304194 37272516 10.1056/NEJMoa2304194 PMC11445763

[32] Mellinghoff IK, Penas-Prado M, Peters KB, Burris HA 3rd, Maher EA, Janku F, Cote GM, de la Fuente MI, Clarke JL, Ellingson BM, Chun S, Young RJ, Liu H, Choe S, Lu M, Le K, Hassan I, Steelman L, Pandya SS, Cloughesy TF, Wen PY (2021) Vorasidenib, a dual inhibitor of mutant IDH1/2, in recurrent or progressive Glioma; results of a first-in-human phase I trial. Clin Cancer Res 27:4491–4499. 10.1158/1078-0432.Ccr-21-0611 34078652 10.1158/1078-0432.CCR-21-0611 PMC8364866

[33] Merchant TE, Hua CH, Shukla H, Ying X, Nill S, Oelfke U (2008) Proton versus photon radiotherapy for common pediatric brain tumors: comparison of models of dose characteristics and their relationship to cognitive function. Pediatr Blood Cancer 51:110–117. 10.1002/pbc.21530 18306274 10.1002/pbc.21530

[34] Meyers CA, Hess KR (2003) Multifaceted end points in brain tumor clinical trials: cognitive deterioration precedes MRI progression. Neuro Oncol 5:89–95. 10.1093/neuonc/5.2.89 12672280 10.1215/S1522-8517-02-00026-1 PMC1920671

[35] Miller JJ (2022) Targeting IDH-mutant Glioma. Neurotherapeutics 19:1724–1732. 10.1007/s13311-022-01238-3 35476295 10.1007/s13311-022-01238-3 PMC9723039

[36] Millward CP, Armstrong TS, Barrington H, Brodbelt AR, Bulbeck H, Byrne A, Dirven L, Gamble C, Grundy PL, Islim AI, Javadpour M, Keshwara SM, Krishna ST, Mallucci CL, Marson AG, McDermott MW, Meling TR, Oliver K, Pizer B, Plaha P, Preusser M, Santarius T, Srikandarajah N, Taphoorn MJB, Watts C, Weller M, Williamson PR, Zadeh G, Zamanipoor Najafabadi AH, Jenkinson MD (2022) Opportunities and challenges for the development of “core outcome sets” in neuro-oncology. Neuro Oncol 24:1048–1055. 10.1093/neuonc/noac062 35287168 10.1093/neuonc/noac062 PMC9248398

[37] Montesinos P, Recher C, Vives S, Zarzycka E, Wang J, Bertani G, Heuser M, Calado RT, Schuh AC, Yeh SP, Daigle SR, Hui J, Pandya SS, Gianolio DA, de Botton S, Döhner H (2022) Ivosidenib and Azacitidine in IDH1-Mutated Acute Myeloid Leukemia. N Engl J Med 386:1519–1531. 10.1056/NEJMoa2117344 35443108 10.1056/NEJMoa2117344

[38] Muroi A, Mizumoto M, Ishikawa E, Ihara S, Fukushima H, Tsurubuchi T, Sakurai H, Matsumura A (2020) Proton therapy for newly diagnosed pediatric diffuse intrinsic pontine glioma. Childs Nerv Syst 36:507–512. 10.1007/s00381-019-04420-9 31728705 10.1007/s00381-019-04420-9

[39] Natsume A, Arakawa Y, Narita Y, Sugiyama K, Hata N, Muragaki Y, Shinojima N, Kumabe T, Saito R, Motomura K, Mineharu Y, Miyakita Y, Yamasaki F, Matsushita Y, Ichimura K, Ito K, Tachibana M, Kakurai Y, Okamoto N, Asahi T, Nishijima S, Yamaguchi T, Tsubouchi H, Nakamura H, Nishikawa R (2023) The first-in-human phase I study of a brain-penetrant mutant IDH1 inhibitor DS-1001 in patients with recurrent or progressive IDH1-mutant Gliomas. Neuro Oncol 25:326–336. 10.1093/neuonc/noac155 35722822 10.1093/neuonc/noac155 PMC9925696

[40] Paganetti H, Zietman A (2015) Why Is Proton Beam Therapy So Controversial? J Am Coll Radiol 12:1318–1319. 10.1016/j.jacr.2015.09.019 26653836 10.1016/j.jacr.2015.09.019

[41] Page MJ, McKenzie JE, Bossuyt PM, Boutron I, Hoffmann TC, Mulrow CD, Shamseer L, Tetzlaff JM, Akl EA, Brennan SE, Chou R, Glanville J, Grimshaw JM, Hróbjartsson A, Lalu MM, Li T, Loder EW, Mayo-Wilson E, McDonald S, McGuinness LA, Stewart LA, Thomas J, Tricco AC, Welch VA, Whiting P, Moher D (2021) The PRISMA 2020 statement: an updated guideline for reporting systematic reviews. BMJ 372:n71. 10.1136/bmj.n71 33782057 10.1136/bmj.n71 PMC8005924

[42] Parsons DW, Jones S, Zhang X, Lin JC, Leary RJ, Angenendt P, Mankoo P, Carter H, Siu IM, Gallia GL, Olivi A, McLendon R, Rasheed BA, Keir S, Nikolskaya T, Nikolsky Y, Busam DA, Tekleab H, Diaz LA Jr, Hartigan J, Smith DR, Strausberg RL, Marie SK, Shinjo SM, Yan H, Riggins GJ, Bigner DD, Karchin R, Papadopoulos N, Parmigiani G, Vogelstein B, Velculescu VE, Kinzler KW (2008) An integrated genomic analysis of human glioblastoma multiforme. Science 321:1807–1812. 10.1126/science.1164382 18772396 10.1126/science.1164382 PMC2820389

[43] Peters KB, Alford C, Heltemes A, Savelli A, Landi DB, Broadwater G, Desjardins A, Johnson MO, Low JT, Khasraw M, Ashley DM, Friedman HS, Patel MP (2024) Use, access, and initial outcomes of off-label ivosidenib in patients with IDH1 mutant Glioma. Neurooncol Pract 11:199–204. 10.1093/nop/npad068 38496920 10.1093/nop/npad068 PMC10940812

[44] Prasad V (2025) Surrogate end points in oncology: the speed-uncertainty trade-off from the patients’ perspective. Nat Rev Clin Oncol. 10.1038/s41571-025-01007-z 40128302 10.1038/s41571-025-01007-z

[45] Pulsifer MB, Sethi RV, Kuhlthau KA, MacDonald SM, Tarbell NJ, Yock TI (2015) Early Cognitive Outcomes Following Proton Radiation in Pediatric Patients With Brain and Central Nervous System Tumors. Int J Radiat Oncol Biol Phys 93:400–407. 10.1016/j.ijrobp.2015.06.012 26254679 10.1016/j.ijrobp.2015.06.012 PMC4955513

[46] Preusser M, Geurts M, Canadian Cancer Trials Group, Cooperative Trials Group for Neuro-Oncology (COGNO) (2025) Vorasidenib maintenance for IDH mutant astrocytoma (VIGOR). ClinicalTrials.gov. https://clinicaltrials.gov/study/NCT06809322. Accessed 30-03-2025

[47] Qiu X, Gao J, Hu J, Yang J, Hu W, Huang Q, Zhang H, Lu JJ, Kong L (2023) Proton radiotherapy in the treatment of IDH-mutant diffuse gliomas: an early experience from shanghai proton and heavy ion center. J Neurooncol 162:503–514. 10.1007/s11060-022-04202-5 36583815 10.1007/s11060-022-04202-5

[48] Retzer A, Baddeley E, Sivell S, Scott H, Nelson A, Bulbeck H, Seddon K, Grant R, Adams R, Watts C, Aiyegbusi OL, Kearns P, Rivera SC, Dirven L, Calvert M, Byrne A (2023) Development of a core outcome set for use in adult primary glioma phase III interventional trials: A mixed methods study. Neurooncol Adv 5:vdad096. 10.1093/noajnl/vdad096 37719788 10.1093/noajnl/vdad096 PMC10503650

[49] Retzer A, Sivell S, Scott H, Nelson A, Bulbeck H, Seddon K, Grant R, Adams R, Watts C, Aiyegbusi OL, Kearns P, Cruz Rivera S, Dirven L, Baddeley E, Calvert M, Byrne A (2022) Development of a core outcome set and identification of patient-reportable outcomes for primary brain tumour trials: protocol for the COBra study. BMJ Open 12:e057712. 10.1136/bmjopen-2021-057712 36180121 10.1136/bmjopen-2021-057712 PMC9528585

[50] Ritterbusch R, Halasz LM, Graber JJ (2021) Distinct imaging patterns of pseudoprogression in glioma patients following proton versus photon radiation therapy. J Neurooncol 152:583–590. 10.1007/s11060-021-03734-6 33751335 10.1007/s11060-021-03734-6

[51] Rydén I, Carstam L, Gulati S, Smits A, Sunnerhagen KS, Hellström P, Henriksson R, Bartek J Jr, Salvesen Ø, Jakola AS (2020) Return to work following diagnosis of low-grade Glioma: a nationwide matched cohort study. Neurology 95:e856–e866. 10.1212/wnl.0000000000009982 32540938 10.1212/WNL.0000000000009982 PMC7605502

[52] Sagberg LM, Salvesen Ø, Jakola AS, Thurin E, De Dios E, Nawabi NLA, Kilgallon JL, Bernstock JD, Kavouridis VK, Smith TR, Solheim O (2024) Progression-free survival versus post-progression survival and overall survival in WHO grade 2 gliomas. Acta Oncol 63:798–804. 10.2340/1651-226x.2024.40845 39428639 10.2340/1651-226X.2024.40845 PMC11500610

[53] Sanai N, Chang S, Berger MS (2011) Low-grade Gliomas in adults. J Neurosurg 115:948–965. 10.3171/2011.7.Jns101238 22043865 10.3171/2011.7.JNS101238

[54] Sankyo D (2020) A Study of DS-1001b in Patients With Chemotherapy- and Radiotherapy-Naive IDH1 Mutated WHO Grade II Glioma. ClinicalTrials.gov. https://clinicaltrials.gov/study/NCT04458272. Accessed 27 Feb 2025

[55] Shih HA, Sherman JC, Nachtigall LB, Colvin MK, Fullerton BC, Daartz J, Winrich BK, Batchelor TT, Thornton LT, Mancuso SM, Saums MK, Oh KS, Curry WT, Loeffler JS, Yeap BY (2015) Proton therapy for low-grade gliomas: Results from a prospective trial. Cancer 121:1712–1719. 10.1002/cncr.29237 25585890 10.1002/cncr.29237 PMC12895348

[56] Slevin F, Hudson EM, Boele FW, Powell JR, Noutch S, Borland M, Brown S, Bruce A, Bulbeck H, Burnet NG, Chang YC, Colaco R, Currie S, Egleston D, Fersht N, Klein M, Lilley J, Lowe M, Miles E, Murray RD, O’Hara DJ, Norris M, Parbutt C, Smith A, Smith C, Whitfield GA, Short S, Murray L (2025) APPROACH: Analysis of Proton versus Photon Radiotherapy in Oligodendroglioma and Assessment of Cognitive Health - study protocol paper for a phase III multicentre, open-label randomised controlled trial. BMJ Open 15:e097810. 10.1136/bmjopen-2024-097810 40010843 10.1136/bmjopen-2024-097810 PMC11865786

[57] Tabrizi S, Yeap BY, Sherman JC, Nachtigall LB, Colvin MK, Dworkin M, Fullerton BC, Daartz J, Royce TJ, Oh KS, Batchelor TT, Curry WT, Loeffler JS, Shih HA (2019) Long-term outcomes and late adverse effects of a prospective study on proton radiotherapy for patients with low-grade glioma. Radiother Oncol 137:95–101. 10.1016/j.radonc.2019.04.027 31082632 10.1016/j.radonc.2019.04.027 PMC6642836

[58] Taphoorn MJ (2003) Neurocognitive sequelae in the treatment of low-grade gliomas. Semin Oncol 30:45–48. 10.1053/j.seminoncol.2003.11.023 14765385 10.1053/j.seminoncol.2003.11.023

[59] Teng KX, Price B, Joshi S, Alukaidey L, Shehab A, Mansour K, Toor GS, Angliss R, Drummond K (2021) Life after surgical resection of a low-grade glioma: A prospective cross-sectional study evaluating health-related quality of life. J Clin Neurosci 88:259–267. 10.1016/j.jocn.2021.03.038 33992194 10.1016/j.jocn.2021.03.038

[60] Thurin E, Nyström PW, Smits A, Werlenius K, Bäck A, Liljegren A, Daxberg EL, Jakola AS (2018) Proton therapy for low-grade gliomas in adults: a systematic review. Clin Neurol Neurosurg 174:233–238. 10.1016/j.clineuro.2018.08.003 30292166 10.1016/j.clineuro.2018.08.003

[61] Tom MC, Cahill DP, Buckner JC, Dietrich J, Parsons MW, Yu JS (2019) Management for different Glioma subtypes: are all low-grade Gliomas created equal? Am Soc Clin Oncol Educ Book 39:133–145. 10.1200/edbk_238353 31099638 10.1200/EDBK_238353

[62] Tricco AC, Lillie E, Zarin W, O’Brien KK, Colquhoun H, Levac D, Moher D, Peters MDJ, Horsley T, Weeks L, Hempel S, Akl EA, Chang C, McGowan J, Stewart L, Hartling L, Aldcroft A, Wilson MG, Garritty C, Lewin S, Godfrey CM, Macdonald MT, Langlois EV, Soares-Weiser K, Moriarty J, Clifford T, Tunçalp Ö, Straus SE (2018) PRISMA extension for Scoping Reviews (PRISMA-ScR): checklist and explanation. Ann Intern Med 169:467–473. 10.7326/m18-0850 30178033 10.7326/M18-0850

[63] van den Bent MJ, Afra D, de Witte O, Ben Hassel M, Schraub S, Hoang-Xuan K, Malmström PO, Collette L, Piérart M, Mirimanoff R, Karim AB (2005) Long-term efficacy of early versus delayed radiotherapy for low-grade astrocytoma and oligodendroglioma in adults: the EORTC 22845 randomised trial. Lancet 366:985–990. 10.1016/s0140-6736(05)67070-5 16168780 10.1016/S0140-6736(05)67070-5

[64] Walia A, Tuia J, Prasad V (2023) Progression-free survival, disease-free survival and other composite end points in oncology: improved reporting is needed. Nat Rev Clin Oncol 20:885–895. 10.1038/s41571-023-00823-5 37828154 10.1038/s41571-023-00823-5

[65] Wick A, Bähr O, Schuler M, Rohrberg K, Chawla SP, Janku F, Schiff D, Heinemann V, Narita Y, Lenz HJ, Ikeda M, Ando Y, Wick W, Steinbach JP, Burger MC, Wenger K, Lassen U, Sankhala KK, Roggia C, Genvresse I, Munhoz C, Rentzsch C, Reschke S, Langer S, Wagner M, Kaulfuss S, Cai C, Lagkadinou E, Jeffers M, Peña C, Tabatabai G (2021) Phase I assessment of safety and therapeutic activity of BAY1436032 in patients with IDH1-mutant solid tumors. Clin Cancer Res 27:2723–2733. 10.1158/1078-0432.Ccr-20-4256 33622704 10.1158/1078-0432.CCR-20-4256

[66] Winter SF, Gardner MM, Karschnia P, Vaios EJ, Grassberger C, Bussière MR, Nikolic K, Pongpitakmetha T, Ehret F, Kaul D, Boehmerle W, Endres M, Shih HA, Parsons MW, Dietrich J (2024) Unique brain injury patterns after proton vs photon radiotherapy for WHO grade 2–3 gliomas. Oncologist 29:e1748–e1761. 10.1093/oncolo/oyae195 39126664 10.1093/oncolo/oyae195 PMC11630789

[67] Yavas C, Zorlu F, Ozyigit G, Gurkaynak M, Yavas G, Yuce D, Cengiz M, Yildiz F, Akyol F (2012) Prospective assessment of health-related quality of life in patients with low-grade glioma: a single-center experience. Support Care Cancer 20:1859–1868. 10.1007/s00520-011-1288-4 21979904 10.1007/s00520-011-1288-4

[68] Yu H, He S, He Y, Dai G, Fu Y, Zeng X, Liu M, Ai P (2024) Dosimetric comparison of advanced radiation techniques for scalp-sparing in low-grade gliomas. Strahlenther Onkol 200:785–796. 10.1007/s00066-024-02229-3 38649484 10.1007/s00066-024-02229-3

