# Supplementary material for: A scoping review of proton radiation therapy and mutant-isocitrate dehydrogenase-inhibitors in IDH mutated lower-grade gliomas: pushing beyond surrogate end-points
Source: Acta Neurochir (Wien). 2025 Jul 19;167(1):196. doi: 10.1007/s00701-025-06612-6 (PMC12276128; doi:10.1007/s00701-025-06612-6)
Supplement: Supplementary file 1 — (PDF 97.0 KB) [file 701_2025_6612_MOESM1_ESM.pdf]

A scoping review of proton radiation therapy and mutant-isocitrate dehydrogenase-inhibitors in IDH mutated lower-grade gliomas: pushing beyond surrogate end-points, ***Acta Neurochirurgica*, Authors:**

Dima Harba, Alba Corell, Alireza Mansouri, Petter Brandal, Malin Blomstrand, Asgeir Store Jakola·
Corresponding Author: Asgeir Store Jakola (jakola.asgeir@gu.se), Institute of Neuroscience and Physiology, Section of Clinical Neuroscience, Sahlgrenska Academy, Gothenburg, Sweden. Department of Neurosurgery, Sahlgrenska University Hospital, Gothenburg, Sweden.413 45 Gothenburg, Sweden

Supplementary Information 1

Search blocks applied in PubMed and Scopus on December 3, 2024. These search blocks were based on terms and keywords used in the Medical Subject Headings (MeSH) thesaurus and existing literature on PRT and mIDH-inhibitors.

-Scopus:

TITLE-ABS-KEY((“low grade glioma” OR astrocytoma OR oligodendroglioma OR idh1 OR idh2 OR LGG OR dLGG OR ”isocitrate dehydrogenase 1” OR ”isocitrate dehydrogenase 2” OR IDH OR ”mutant lower grade glioma”)

AND

(Proton OR ”Proton therap*” OR ”proton radiotherapy*” OR ”proton beam*” OR ”proton radiation*” OR ”Isocitrate Dehydrogenase” OR ”Isocitrate Dehydrogenase inhibitor” OR ”IDH-mutation inhibitor” OR ”IDH inhibitor” OR vorasidenib OR olutasidenib OR ivosidenib OR ivosidenib OR ”BAY 1436032” OR BAY1436032 OR DS-1001 OR LY3410738))

-PubMed:

(low grade glioma[tiab] OR astrocytoma[tiab] OR oligodendroglioma[tiab] OR idh1[tiab] OR idh2[tiab] OR LGG[tiab] OR dLGG[tiab] OR isocitrate dehydrogenase 1[tiab] OR isocitrate dehydrogenase 2[tiab] OR ((IDH[tiab] OR IDH1[tiab] OR IDH2[tiab]) AND (mutant lower grade glioma*[tiab])))

AND

(Proton[tiab] OR Proton therapy[mesh] OR proton therap*[tiab] OR proton radiotherapy*[tiab] OR proton beam[tiab] OR proton radiation*[tiab] OR Isocitrate Dehydrogenase[mesh] OR Isocitrate Dehydrogenase inhibitor*[tiab] OR IDH-mutation inhibitor*[tiab] OR IDH inhibitor*[tiab] OR vorasidenib[Supplementary Concept] OR vorasidenib[tiab] OR olutasidenib[Supplementary Concept] OR olutasidenib[tiab] OR ivosidenib[Supplementary Concept] OR ivosidenib[tiab] OR BAY 1436032[Supplementary Concept] OR BAY1436032[tiab] OR DS-1001[tiab] OR LY3410738[tiab])
